# Novel markers in pediatric-type follicular lymphoma

**DOI:** 10.1007/s00428-019-02681-y

**Published:** 2019-11-04

**Authors:** Claudio Agostinelli, Ayse U Akarca, Alan Ramsay, Hasan Rizvi, Manuel Rodriguez-Justo, Sabine Pomplun, Ian Proctor, Elena Sabattini, David Linch, Stephen Daw, Stefania Pittaluga, Stefano A Pileri, Elaine S Jaffe, Leticia Quintanilla-Martinez, Teresa Marafioti

**Affiliations:** 1 Haematopathology Unit, Department of Experimental Diagnostic and Specialty Medicine, S. Orsola-Malpighi Hospital, University of Bologna, Bologna, Italy; 2 Department of Pathology, University College London, London, UK; 3 Department of Cellular Pathology, University College Hospital London, London, UK; 4 Department of Cellular Pathology, Barts Health NHS Trust, London, UK; 5 Department of Haematology, University College London Cancer Institute, London, UK; 6 Children and Young People’s Cancer Service, University College Hospital London, London, UK; 7 Haematology section, Laboratory of Pathology, Center for Cancer Research National Cancer Institute, Bethesda, MD USA; 8 Division of Haematopathology, European Institute of Oncology, University Hospital of Tübingen, Institute of Pathology, Tübingen, Germany; 9 University Hospital of Tübingen, Institute of Pathology, Tübingen, Germany

**Keywords:** Pediatric-type follicular lymphoma, Immunohistochemistry, Differential diagnosis, FOXP-1

## Abstract

The aim of this study was to review the histopathological, phenotypic, and molecular characteristics of pediatric-type follicular lymphoma (PTFL) and to assess the diagnostic value of novel immunohistochemical markers in distinguishing PTFL from follicular hyperplasia (FH). A total of 13 nodal PTFLs were investigated using immunohistochemistry, fluorescence in situ hybridization (FISH), and PCR and were compared with a further 20 reactive lymph nodes showing FH. Morphologically, PTFL cases exhibited a follicular growth pattern with irregular lymphoid follicles in which the germinal centers were composed of numerous blastoid cells showing a starry-sky appearance. Immunohistochemistry highlighted preserved CD10 (13/13) and BCL6 (13/13) staining, CD20 (13/13) positivity, a K light chain predominance (7/13), and partial BCL2 expression in 6/13 cases (using antibodies 124, E17, and SP66). The germinal center (GC)–associated markers stathmin and LLT-1 were positive in most of the cases (12/13 and 12/13, respectively). Interestingly, FOXP-1 was uniformly positive in PTFL (12/13 cases) in contrast to reactive GCs in FH, where only a few isolated positive cells were observed. FISH revealed no evidence of *BCL2*, *BCL6*, or *MYC* rearrangements in the examined cases. By PCR, clonal immunoglobulin gene rearrangements were detected in 100% of the tested PTFL cases. Our study confirmed the unique morphological and immunophenotypic features of PTFL and suggests that FOXP-1 can represent a novel useful diagnostic marker in the differential diagnosis between PTFL and FH.

## Introduction

Pediatric-type follicular lymphoma (PTFL) is a distinct clinicopathological entity in the revised 4th edition of the WHO Classification of Tumours of Haematopoietic and Lymphoid Tissues [1].

Although PTFL was initially identified in pediatric patients, accounting for approximately 1–2% of all pediatric non-Hodgkin lymphomas (NHL), it was subsequently found to occur also in young adults and more rarely in older patients [1–12]. The median age at onset ranges from 7.5 to 14 years, and the disease shows a male predominance (male to female ratio > 10:1) [1–12]. PTLF is currently regarded as a nodal disease that most commonly arises in head and neck lymph nodes and less frequently involves inguinal and axillary nodes [1, 10, 11]. Because of differences in histopathologic features, molecular profile, and clinical behavior, the revised 4th edition of WHO classification excluded from this category extranodal follicular lymphoma (FL) cases involving testis, epididymis, gastrointestinal tract, and kidney, which had been previously reported as PTFL [1]. Another entity that should be excluded is the new provisional entity “large B-cell lymphoma (LBCL) with *IRF4* rearrangement,” [1, 13–15] which usually presents in Waldeyer’s ring and/or cervical lymph nodes but can also arise in the gastrointestinal tract; this lesion may be exclusively follicular, follicular and diffuse, or diffuse.

Most PTFL patients present with localized disease and after local excision show complete remission with excellent prognosis and disease-free survival; for this reason, a “watch and wait” strategy is currently recommended [1–9]. Histologically, the neoplastic follicles in PTFL are large and irregularly expanded, sometimes coalescent and are highly proliferative with prominent tingible body macrophages. Although largely meeting the current histological criteria for conventional grade 3B FL, a proportion of cases lack classical centroblasts and centrocytes and consist instead of medium-sized blastoid cells [1]. The revised 4th WHO classification indicates that *BCL2* rearrangements are not present in PTFL [1]; however, BCL2 protein expression has been reported in a minority of cases, usually with weak intensity [1]. PTFL also lacks *BCL6*, *IRF4*, and *MYC* rearrangements [1, 10, 11]. The molecular profile of PTFL differs from that of conventional t(14;18)^+^ and t(14;18)^−^ FL, as PTFL is characterized by a low genomic complexity and lacks or has only rare mutations in the histone-modifying genes *CREBBP*, *EZH2*, and *KMT2D*, commonly found in conventional FL [10–12]. Furthermore, *TNFRSF14* and *MAP2K1* mutations are the most frequently reported genetic aberrations in PTFL [10–12]. A hot spot mutation in *IRF8* (K66R, p.L66A), although less frequently reported, seems unique in PTFL [16]. In spite of the present knowledge of this condition, some cases present a challenge in the differential diagnosis with florid follicular hyperplasia (FH), pediatric nodal marginal zone lymphoma (NMZL), and other t(14;18)^−^ FLs. Our purpose was to undertake a histopathological review and perform phenotypic and molecular analyses of a series of PTFLs, to assess the potential diagnostic value of novel markers which could assist in differentiating PTFL from FH.

## Material and methods

### Tissue samples

Formalin-fixed, paraffin-embedded (FFPE) tissue blocks of 37 cases originally diagnosed as PTFL were retrieved from the files of the Department of Histopathology, University College Hospital, London (UK); Department of Pathology, Birmingham (UK); and the Unit of Haematopathology, S. Orsola-Malpighi Hospital, University of Bologna (Italy). In addition, 20 cases of reactive lymph nodes with FH of children and young adults diagnosed at the Department of Histopathology, University College Hospital London, were included in the study as controls.

The 37 PTFL cases were reviewed by expert hematopathologists (TM, LQF, SAP, ESJ). A consensus diagnosis of PTFL was reached in 13 out of the 37 cases, by strict adherence to the following criteria of the revised WHO classification: (a) nodal disease, (b) pure follicular growth pattern with lack of diffuse areas, (c) morphology characterized by large expansile highly proliferative follicles often consisting of blastoid germinal center cells rather than classic centroblasts or centrocytes, (d) BCL6 expression with associated BCL2 negativity or weak positivity and high proliferative fraction (> 30%) by immunohistochemistry, (e) absence of *BCL2*, *BCL6*, and *MYC* rearrangements as well as *BCL2* amplifications [1]. To further confirm the neoplastic nature of the process, at least one of the following parameters was required: detection of IGH and/or IGK gene rearrangements. Cases characterized by IRF4^+^ follicles, in the absence of a negative FISH analysis of the corresponding gene, were excluded from the study to avoid possible inclusion of cases of LBCL with *IRF4* rearrangement.

### Antibodies and immunohistochemistry

Antibodies raised against fixation resistant epitopes were used for the detection of CD20 (mouse, clone L26, Dako, Ely, UK), CD3 (mouse, clone LN10, Leica Microsystems, Newcastle-upon-Tyne, UK), CD10 (mouse, clone 56C6, Leica Microsystems, Newcastle-upon-Tyne, UK), BCL6 (mouse, clone GI191E/A8, CNIO, Madrid), BCL2 (mouse, clone 124, Dako, Ely, UK), BCL2 (Rabbit, clone E17, Menarini Diagnostics, Wokingham, UK), BCL2 (rabbit, clone SP66, Spring Bioscience, Pleasanton, CA, USA), IRF4/MUM1 (mouse, clone MUM1p, kindly provided by Prof. Brunangelo Falini, Perugia, Italy), IRTA-1 (mouse monoclonal, kindly provided by Prof. Brunangelo Falini, Perugia, Italy), c-MYC (rabbit, clone Y69, Epitomics), IgM (mouse polyclonal, Dako A/S, Glostrup, Denmark), IgD (mouse polyclonal, Dako A/S, Glostrup, Denmark), kappa (mouse polyclonal, Dako A/S, Glostrup, Denmark) and lambda (mouse polyclonal, Dako A/S, Glostrup, Denmark) light chains, CD21 (mouse, clone 1F8, Dako A/S, Glostrup, Denmark), forkhead box protein P1 (FOXP-1) (mouse, clone JC12 AbD Serotec, Oxford, UK), stathmin (STMN1) (rabbit, clone SP49, Spring Bioscience, Pleasanton, CA, USA), lectin-like transcript 1 (LLT1) (goat polyclonal, R&D Systems), and Ki-67/MIB1 (mouse, clone MIB1, Dako, Ely, UK). Immunostaining was performed using a BOND-III AutoStainer (Leica Microsystems, Newcastle-upon-Tyne, UK) as previously described [17]. A cutoff of staining > 30% of the examined cells was assigned as positive score, according to formerly defined criteria [18].

### FISH

Interphase fluorescent in situ hybridization (FISH) for the detection of *BCL2*, *BCL6*, *IRF4*, and *MYC* chromosomal alterations was performed using Vysis (Abbott Italia, Rome, Italy) LSI *BCL2* dual color, break apart rearrangement probe (18q21); Vysis LSI *BCL6* (ABR) dual color, break apart rearrangement probe (3p27); Kreatech (Leica Microsystems, Buccinasco, Milano, Italy) *IRF4* dual color, break apart rearrangement probe (6p25.3); and Vysis LSI *MYC* dual color, break apart rearrangement probe (8q24) (all Abbott, Abbott Park, IL, USA). The cutoff value for the diagnosis of each probe set was the mean percentage of cells with a false-positive signal constellation plus 3 standard deviations, as assessed on tissue from reactive lymph nodes. The break apart/split signal rearrangement probes also detect loss or amplification of the respective genes. FISH procedure was performed according to published standard methods [19, 20].

### DNA extraction and clonality analysis

Sections of 5-μm thick were prepared from paraffin-embedded biopsy specimens. Samples were first deparaffinized in histoclear and then rehydrated in ethanol. Tissue digestion was carried out in 200 μl of a solution containing 20 μl of proteinase K in 180 μl ATL Buffer (Qiagen); subsequently, the material was incubated at 56 °C overnight. Finally, DNA purification was performed by using a Qiagen DNA mini kit according to the manufacturer’s instructions. DNA sample concentration and quality were assessed by spectrophotometry (260/280 nm using the NanoDrop). Only cases with a 260/280 nm ratio between 1.8 and 2 and 260/230 ratio of about 2.2 were considered evaluable. IGH and IGK gene rearrangements were analyzed by multiplex polymerase chain reaction (PCR) amplification according to the BIOMED-2 multiplex PCR protocol as previously reported [21].

## Results

### Patients

According to the criteria of the revised WHO classification [1], 13 out the original 37 cases were included in the study as PTFLs along with 20 FHs. After clinical, pathological, and immunophenotypic review, the 24 excluded cases comprised 6 inadequate samples, 8 cases re-classified as pediatric-type marginal zone lymphoma, 5 cases recognized as conventional FL (these cases showed *BCL2* rearrangement), and 2 cases with extranodal localization involving the testis and conjunctiva. Two cases, which the histology and phenotype suggested PTFL, were excluded since neither rearrangement of the IGH and/or IGK genes was detected. An additional case was excluded after immunohistochemical review due to a diffuse and strong expression of IRF4, along with CD10 and BCL6, in the absence of *IRF4* FISH analysis.

All patients included in the study were male, with an age range between 6 and 24 years and a median age of 15 years (Table 1). All cases were nodal in presentation: 6 in the head and neck region, 4 in the inguinal region, and 1 in the axilla (Table 1). For the remaining 2 cases, no specific anatomic site was recorded.

**Table 1:** Immunophenotypic and molecular findings in PTFLs

Case ID	Age	Sex	Biopsy site	Immunophenotype													FISH				PCR
				CD20 13/13^a^ (100)	CD10 13/13 (100)	BCL6 13/13 (100)	BCL2^b^ 6/13 (40)	IRF4^c^ 0/13 (0)	Igk 7/12 (60)	Igλ 5/12 (40)	IgM 9/11 (85)	IRTA1^d^ 2/13 (1)	LLT1 12/13 (87)	STMN1 12/13 (93)	FOXP-1 12/13 (93)	MYC^e^ 0/13 (0)	*BCL2* 0/13 (0)	*BCL6* 0/13 (0)	*MYC* 0/13 (0)	IG*/IRF4* 0/4 (0)	*IGH/k*^f^ 9/9 (67)
PTFL1	15	M	Lymph node	+	+	+	–	–	+	–	+	+	–	+	+	–	–	–	–	–	+
PTFL2	11	M	Peri-auricular lymph node	+	+	+	+	–	–	+	+	–	+	+	+	–	–	–	–	n.d	n.m.a
PTFL3	6	M	Intra-Parotid lymph node	+	+	+	+	–	+	–	+	+	+	+	+	–	–	–	–	–	n.m.a
PTFL4	24	M	Lymph node	+	+	+	–	–	+	–	–	–	+	+	+	–	–	–	–	–	+
PTFL5	8	M	Axillary lymph node	+	+	+	–	–	+	–	+	–	+	+	+	–	–	–	–	–	+
PTFL6	14	M	Submandibular lymph node	+	+	+	–	–	+	–	n.m.a	n.m.a	+	–	–	–	–	–	–	n.d	+
PTFL7	20	M	Submandibular lymph node	+	+	+	+	–	+	–	+	–	+	+	+	–	–	–	–	n.d	n.e.
PTFL8	17	M	Inguinal lymph node	+	+	+	+	–	n.m.a	n.m.a	n.m.a	–	+	+	+	–	–	–	–	n.d	+
PTFL9	18	M	Cervical lymph node	+	+	+	–	–	–	+	+	–	+	+	+	–	–	–	–	n.d	+
PTFL10	19	M	Inguinal lymph node	+	+	+	+	–	–	+	+	–	+	+	+	–	–	–	–	n.d	n.e.
PTFL11	12	M	Inguinal lymph node	+	+	+	–	–	–	+	+	–	+	+	+	–	–	–	–	n.d	+
PTFL12	17	M	Cervical lymph node	+	+	+	–	–	–	+	+	–	+	+	+	–	–	–	–	n.d	+
PTFL13	15	M	Inguinal lymph node	+	+	+	+	–	+	–	–	–	+	+	+	–	–	–	–	n.d	+

^a^Number of positive/total cases (%)

^b^BCL2 staining was performed using three different clones (124, E17, SP66); in 5 out of the 15 cases, a proportion of atypical cells were BCL-2 positive

^c^In 5 out of 15 cases, focal MUM-1 positivity was observed

^d^IRTA-1 positivity was restricted to areas of morphological marginal zone differentiation

^e^All are negative, but in 10 out of 15 cases, a proportion of the tumor cells (i.e., < 1% up to 10%) were positive

^f^Presence (+) or absence (−) of *IGH* and/or *IGk* genes rearrangement

*n.e.*, not evaluable, in PTFL7 the FR1 region of *IGH* and *IGK* resulted not evaluable and in PTFL12 FR1 and FR2 region of *IGH* were not evaluable; *n.d*, not done; *n.m.a*, no more material available for further analysis

### Histological and immunophenotypic findings

**Fig. 1:**
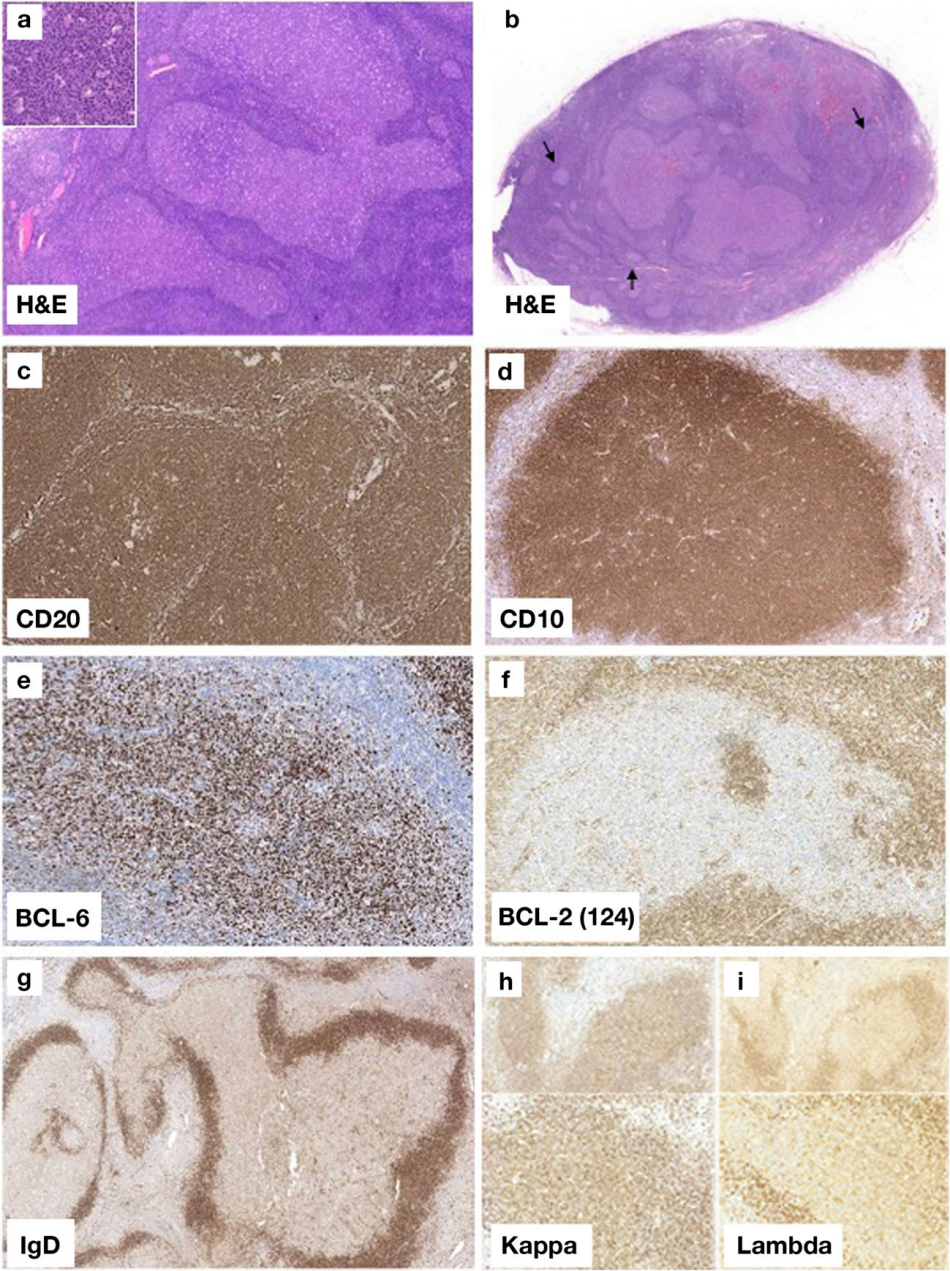
**a** PTFL in a lymph node showing the characteristic expanded, irregular, and often closely packed lymphoid follicles with serpiginous outlines and attenuated mantle zones (hematoxylin and eosin; × 10). The GCs contain a monotonous proliferation of medium- to large-sized blastoid cells with round/oval nuclei, finely clumped chromatin, and small nucleoli (inset; hematoxylin and eosin; × 40). **b** The black arrows indicate small reactive follicles adjacent to the follicular proliferation. This low-power image illustrates the so-called “a node within a node” appearance (hematoxylin and eosin; × 2). **c** The neoplastic follicles are strongly and homogeneously positive for CD20, CD10 (**d**), and BCL6 (**e**) (× 200). **f** The GCs in PTFL lack strong BCL2 protein expression (× 200). **g** The enlarged follicles show attenuated IgD-positive mantle zones (× 200). **h**, **i** Light chain staining of an abnormal germinal center in PTFL showing positive staining for kappa light chains (**h**) and negative lambda staining (**i**) (at both × 20 and × 200 magnifications)

The 13 selected PTFLs showed a purely follicular growth pattern with irregular, often closely packed lymphoid follicles, many of which exhibited serpiginous outlines and attenuated mantle zones (Fig. 1a). The GCs lacked normal polarization while retaining a starry-sky appearance, and consisted of a monotonous proliferation of medium- to large-sized blastoid cells with round/oval nuclei, finely clumped chromatin, and small nucleoli (Fig. 1a inset) as distinct from typical centrocytes and centroblasts. Where intact lymph nodes were sampled, it was not uncommon to see small reactive follicles at the periphery of the specimen; the appearances of these two distinct areas, reactive follicles and enlarged abnormal follicles, result in the so called “node within a node” pattern (Fig. 1b). Extra capsular extension of neoplastic follicles was not observed. None of the cases showed areas with a diffuse growth pattern, although focal confluent follicles were noted. In some cases, a narrow peripheral rim of paler cells resembling “monocytoid differentiation” was seen around the expanded follicles; however, areas of true monocytoid B cell hyperplasia were lacking.

On immunophenotyping, the neoplastic follicles in PTFL were strongly and homogeneously CD20^+^ (13/13), CD10^+^ (13/13), and BCL6^+^ (13/13), with very few or no atypical cells in the interfollicular areas (Table 1) (Fig. 1c–e). BCL2 protein expression was seen in 6/13 (46%) cases, although the staining was weak and heterogeneous, varying in intensity between different follicles (Fig. 1f). None of the PTFL cases demonstrated expression of the MYC protein (Table 1). Ki-67 showed a high proliferation fraction often with accentuation at the periphery of the GCs. IgD and CD21 highlighted the attenuated mantle zones and expanded irregular, though not fragmented, follicular dendritic cell (FDC) meshworks respectively (Fig. 1g). Staining for immunoglobulin heavy and light chains was carried out in most cases (in few instances, no additional sections could be cut due to tissue exhaustion). The results showed expression of intracytoplasmic IgM in 9/11 (81%) cases, and kappa or lambda light-chain restriction in 7/12 (60%) and 5/12 (40%), respectively (Fig. 1h, i).

**Fig. 2:**
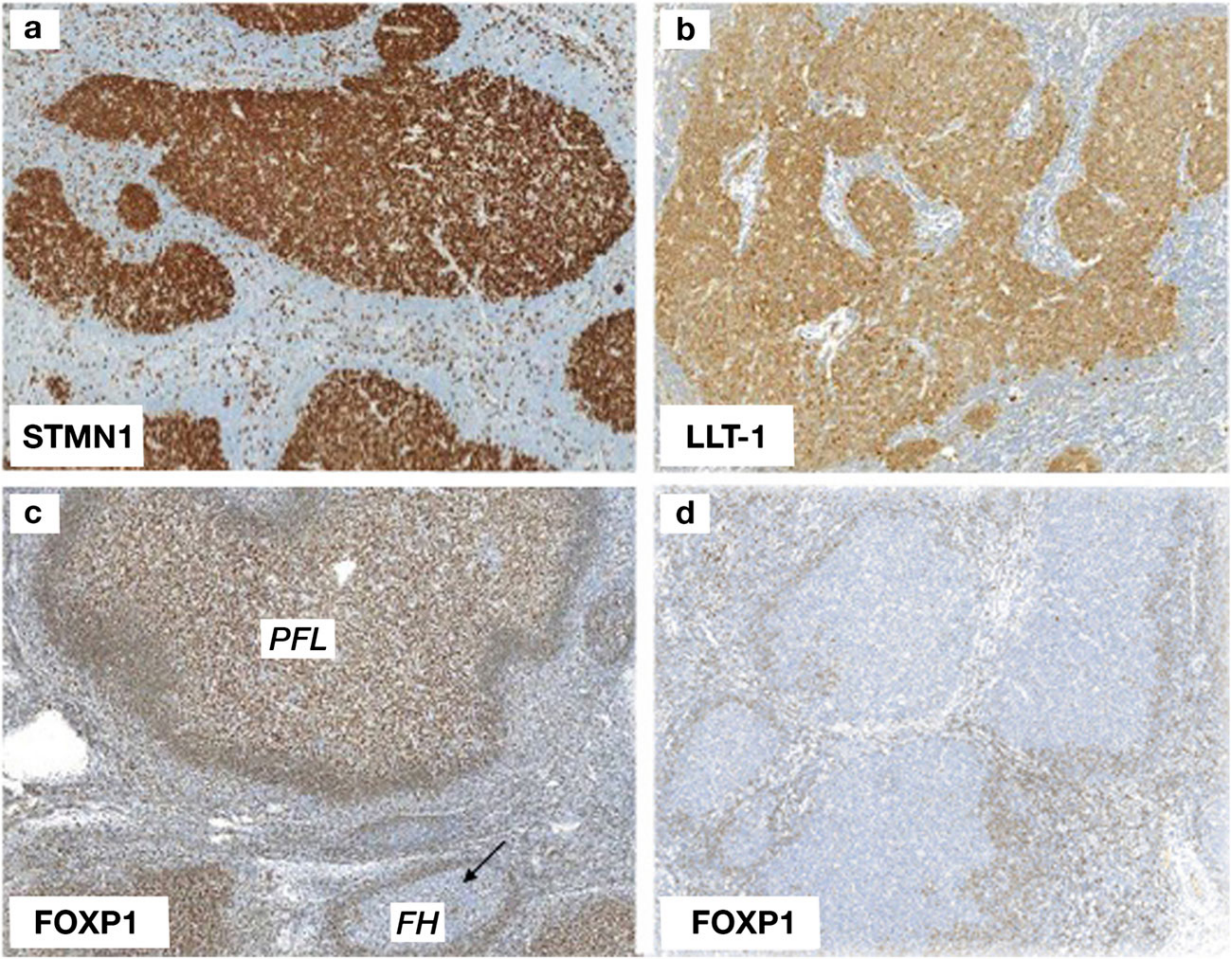
Neoplastic GCs in PTFL expressing **a** STMN-1 (× 100) and **b** LLT-1 (× 100). **c** Strong nuclear staining for FOXP-1 in PTFL germinal centers. The black arrow highlights an included reactive GC which is negative with this marker (× 100). **d** Lymph node from a case of FH. The expanded germinal centers are negative for FOXP-1 (× 100)

The germinal center–associated molecules LLT-1 and STMN-1 were both expressed in 12/13 (92%) (Fig. 2a, b); the STMN-1-negative (PTFL6) and the LLT-1-negative (PTFL1) cases both showed a monoclonal IG gene rearrangement. The marginal zone marker IRTA-1 was focally detected in 2/13 (15%) of the cases in areas of “monocytoid differentiation” at the periphery of follicles. Strong nuclear staining for the transcription factor FOXP-1 in > 80% cells was found in 12/13 (92%) of PTFLs (Fig. 2c). The FOXP-1-negative case (PTCL6) carried a clonal rearrangement of the IG genes.

In contrast, investigation of FOXP-1 expression in the 20 lymph nodes with FH showed that no cases exhibited FOXP-1 positivity in the reactive germinal centers (Fig. 2d).

### Genetics

In eleven cases, the IG rearrangement status was investigated by PCR. In 9/9 patients, IGH and/or IGK gene rearrangements were detected. In PTFL7 and PTFL10, IG analysis yielded suboptimal amplification, with no defined clonal peaks (the FR1 region of IGH and IGK in PTFL7 and the FR1 and FR2 region of IGH in PTFL10 were not evaluable); however, PTFL7 and PTFL10 showed BCL2^+^/*κ*^+^ and BCL2^+^/*λ*^+^ germinal centers respectively. In the remaining two samples (PTFL2 and PTFL3), no further material for molecular studies was available, but the GCs respectively stained as BCL2^+^/*λ*^+^ and BCL2^+^/*κ*^+^.

FISH performed in 13 cases disclosed no rearrangements of *BCL2*, *BCL6*, and *MYC.* Four cases were investigated also with IGH*/IRF4* fusion probes, but no translocations were detected.

## Discussion

PTFL is an uncommon clinicopathological entity newly recognized in the revised 4th edition of the WHO Classification of Tumours of Haematopoietic and Lymphoid Tissues. It predominantly occurs in young patients and only rarely in the elderly and differs from conventional FL on morphologic, genetic, and clinical grounds [1]. In line with previous reports [2–11], in our series, age at onset ranged between 6 and 24 years with a median age of 15 years. Our study also confirmed the strong association with male gender and the unique histological and phenotypic features of PTFL [2–11].

Recent studies have successfully delineated the molecular profile of PTFL [10–12, 16]. Two independent groups performed a genome-wide analysis demonstrating that when compared with conventional t(14;18)^+^ and t(14;18)^−^ FL, nodal PTFL is characterized by low genomic complexity (which fits well with the extremely indolent clinical behavior) and rare or absence of the characteristic mutations in the epigenetic modifiers *CREBBP*, *EZH2*, and *KMT2D* [10–12]. Recurrent mutations/deletions of *TNFRSF14* and mutations of *MAP2K1* genes were found in 54% and 49% of the samples respectively; these were mutually exclusive in the majority of the cases [10–12]. Furthermore, mutation of the *IRF8* gene was reported in a subset of patients (15%) ^12, 16^. These most frequent genetic aberrations might play a pivotal role in the pathogenesis of the disease, altering pathways associated with immune escape, apoptosis, and the germinal center reaction [10–12].

In a diagnostic setting, the main histological differential diagnoses are florid follicular hyperplasia (FFH) (given the expanded GCs and lack of strong BCL2 expression), pediatric marginal zone lymphoma (PMZL) which shows morphological and clinical similarities, t(14;18)^−^ FL where the age at presentation may overlap, and LBCL with *IRF4* rearrangements. The latter condition occurs in the same age group as PTFL; it often involves Waldeyer’s ring and can be also be seen in head and neck lymph nodes and the gastrointestinal tract, and may follow a more aggressive clinical course [13–15]. One morphological feature that is helpful in differentiating PTFL from FFH is the identification of a monotonous population of blastoid cells in the expanded GCs in PTFL; FFH shows polarized follicles composed of centroblasts and centrocytes [1]. Additionally, the germinal center cells in PTFL show very strong CD10 and BCL6 expression; staining is usually stronger than that seen in the residual GCs at the periphery of the node. These residual GCs contrast with the large irregular GC in PTFL, giving the characteristic “node within a node” appearance. Clonality analysis is mandatory for the diagnosis of PTFL. Accordingly, when evaluable, all our cases showed IGH and/or IGk rearrangement and monotypic restriction for immunoglobulin light chains.

Given the above-mentioned challenges in the diagnosis of PTFL, current study included an assessment of novel biomarkers for potential application in this diagnostic setting. Based on our data, a phenotypic feature that consistently differentiated PTFL from FFH was the expression of the FOX-P1 protein, which we found in most PTFL cases of our series, but not in the cases of FFH. The *FOXP-1* (Forkhead Box P1) gene located at 3p14.1 codes for a homologous transcriptional regulator (FOXP-1), belonging to the FOX transcription factor family, which is implicated in a wide range of biological processes, including B cell development and immune response regulation. FOXP-1 is downregulated in germinal centers, its expression being inversely related to BCL6 expression and also has been reported to repress plasma cell differentiation [22, 23]. It cooperates with NF-kB signaling to promote expansion of primary mature human B cells [24]. In transgenic mice, constitutive FOXP-1 expression impairs GC formation and function, which might contribute to B cell lymphomagenesis [22]. FOXP-1 positivity has been reported in FL, MZL, and primary testicular lymphomas and represents a prognostic marker in a subset of large B cell lymphomas [25–34]. *FOXP-1* overexpression has been reported in association with t(3;14)(p14.1;q32), leading to a *FOXP-1*-IGH fusion and in rearrangements with non-IG partners, causing the expression of the N-truncated isoforms, or in trisomy 3 [25–34]. Recently, Mottok et al. [35] demonstrated that immunohistochemical expression of FOXP-1 predicted adverse failure–free survival in conventional FL treated with immunochemotherapy. They found FOXP-1 to be significantly downregulated in both *EZH2*- and *MEF2B*-mutated cases and that high FOXP-1 expression was associated with distinct molecular features such as *TP53* mutations, expression of IRF4, and gene expression signatures reminiscent of dark zone germinal center or activated B cells [35].

We demonstrated strong nuclear staining in > 80% of cells for the transcription factor FOXP-1 in 93% of our PTFLs while none of the FH cases analyzed showed FOXP-1 positivity in the reactive GCs. The FOXP-1 expression in PTFL was also confirmed at the RNA level by RNAscope in 5 cases (unpublished data). The level of FOXP-1 positivity in PTFL was higher than the one observed by Mottok et al. [35] in conventional FL: they reported 40% of FOXP-1^+^ cases, applying a cutoff positivity of 10%. This discrepancy could be related to differences in sensitivity in the detection systems used in the two studies or might reflect the biological differences between PTFL and FL in adults as previously delineated by Schmidt at al. [10].

Moreover, we identified in PTFL aberrant co-expression of FOXP-1 and BCL6 protein, proteins that are normally reciprocally expressed in the GC [22]. The presence of the genetic aberration(s) sustaining FOXP-1 overexpression in PTFL needs to be investigated in further studies. However, our data supports the use of FOXP-1 as a candidate marker of neoplastic transformation and an essential tool for differentiating PTFL from FH.

The differential diagnosis between PTFL and PMZL can be challenging due to the shared features (male predominance, localized disease, excellent prognosis) [36]. On morphologic grounds, progressively transformed germinal center–like changes and residual hyperplastic GCs maintaining a starry-sky appearance as well as polarization suggest the diagnosis of PMZL [4, 6, 36]. PMZL is also characterized by expanded interfollicular areas consisting of CD20^+^ B cells and the frequent negative immunohistochemical staining for CD10 and BCL6 [4, 6, 36]. The lack of interfollicular involvement by atypical CD20^+^ cells in our cases, which is highly characteristic of PTFL, facilitated the distinction from PMZL. Furthermore, most nodal and extranodal marginal zone lymphomas express IRTA-1^+^ [37], a protein only focally detected in 2/13 (15%) of our PTFLs where there were features indicating a degree of marginal zone differentiation. The diagnosis of PTFL was also favored by the expression of the GC-associated molecules STMN1 and LLT-1 in 12/13 (92%) and in 12/13 (92%) of the cases respectively. STMN1 is an intracellular protein involved in multiple cell signaling pathways and is a GC-associated marker [38]. Previous studies of STMN1 expression in solid and hematological malignancies have shown an association with tumor behavior and prognosis [39–42]. In addition, we previously demonstrated the diagnostic relevance of STMN1 for FLs including those that are BCL2 negative, and showed its potential usefulness in distinguishing FLs from marginal zone lymphomas (MZLs), which are usually STMN1 negative [38].

LLT-1 is known to be present in GC-associated B cells and early plasmablasts. Its expression is readily induced via BCR, CD40, and CpG stimulation on B cells. The LLT1 ligand, CD161, was found to be highly expressed on follicular dendritic cells suggesting that LLT1-CD161 interactions play a novel and important role in B cell maturation [43]. LLT1 expression was seen on GC-derived lymphomas including Burkitt lymphoma (73%), conventional FL (51%), and lymphocyte predominant Hodgkin lymphoma (44%) ^43^. Our data suggest that STMN1 and LLT1 are maintained during malignant transformation in cases of PTFL and may be useful additional diagnostic markers in the differential diagnosis between PTFL and PMZL.

In summary, in this study, we reported a series of PTFL cases and demonstrated that the expression of novel GC-associated markers LLT-1 and STMN-1 is helpful in the differential diagnosis with PMZL. Additionally, we demonstrated that in contrast to FH, FOXP-1 is consistently expressed in the germinal centers in PTFL. Since FOXP-1 is not expressed in reactive GC, it emerges as a new diagnostic marker for PTFL and is particularly helpful in differentiating this condition from florid FH.
